# Calcified Foley Catheter and Vesicovaginal Fistula: A Case Report

**DOI:** 10.7759/cureus.106567

**Published:** 2026-04-07

**Authors:** Kramo N Felicite, Drabo Ali, Coulibaly Issoufou, Konan Kevin, Gnabro Alain

**Affiliations:** 1 Urology, Université Félix Houphouët-Boigny, Abidjan, CIV

**Keywords:** bladder stones, embedded catheter, foley’s catheter, urine incontinence, vesicovaginal fistula

## Abstract

Calcification of indwelling urinary catheters is a known complication of prolonged urinary catheterization. It is caused by the formation of a bacterial biofilm, which promotes the accumulation of calcium deposits around the balloon. These deposits can lead to recurrent urinary tract infections, bladder stones, and, more rarely, bladder wall damage that can progress in the long term to a vesicovaginal fistula.

We report the case of a 56-year-old woman living in a rural area who presented with persistent vaginal urinary incontinence. The patient reported having a urinary catheter that had been left in place for three years without being replaced, and whose distal portion had been severed by the patient herself in an attempt at self-removal. Urogenital examination revealed a calcified Foley catheter associated with a vesicovaginal fistula. Treatment consisted of transection of the distal portion of the catheter via the urethra, followed by extraction of the intravesical fragment and calculi through the fistulous opening. The fistula was repaired vaginally during the same procedure. The postoperative course was uneventful, with recovery of urinary continence upon catheter removal on day 21 and no recurrence at postoperative follow-up.

This observation describes a rare complication of prolonged urinary catheterization and underlines the importance of regular follow-up and education of patients with long-term urinary catheters.

## Introduction

Urinary catheterization has been used since antiquity to ensure bladder drainage [1]. The Foley catheter was introduced in the 1930s to allow continuous drainage and ensure hemostasis in prostate surgeries [2]. It very quickly became one of the most widely used medical implant devices in the management of bladder dysfunction in many patients [3]. Despite its usefulness, several complications have been associated with its prolonged use. These include bacterial colonization, obstructions, the appearance of resistant bacterial strains, chronic bladder infection, catheter calcification, and bladder stones [2-4]. Chronic irritation and pressure from a calcified catheter can lead to bladder wall damage, ischemia, and tissue necrosis, potentially progressing to a vesicovaginal fistula. Although this mechanism has been described, this complication remains rare [5].

We report the case of a 56-year-old patient with an unaltered indwelling catheter of approximately three years that became calcified, leading to the formation of a vesicovaginal fistula. Catheter fragments and calculi were removed through the fistulous opening; the fistula was repaired during the same surgical procedure.

## Case presentation

A 56-year-old female patient, a housewife living in a rural area, consulted for a permanent leakage of urine from the vagina.

She has been menopausal for almost 10 years. Her medical history included urinary problems that had been evolving for several years after menopause, including stress urinary incontinence, urgency, and frequency. She consulted several health centers where medical treatments were initiated without improvement. 

According to the patient, an undocumented vaginal surgery was performed, probably for urinary incontinence, due to the failure of medical treatment. Despite this procedure, the symptoms persisted. A so-called comfort urinary catheter, to be replaced monthly, was then inserted for an initially planned duration of three months, with cessation of urinary leakage as long as the catheter was in place.

Due to financial constraints and geographical distance, the patient did not attend her last appointment for catheter removal and renewal. 

She then attempted to remove the catheter herself, but cut it, leaving the intravesical segment in place for three years. Initially, her condition was marked by peri-catheter urine leakage, followed, after approximately two years, by the development of persistent vaginal urine leakage, causing her to seek treatment at a free mobile clinic for urogenital fistulas.

**Figure 1:**
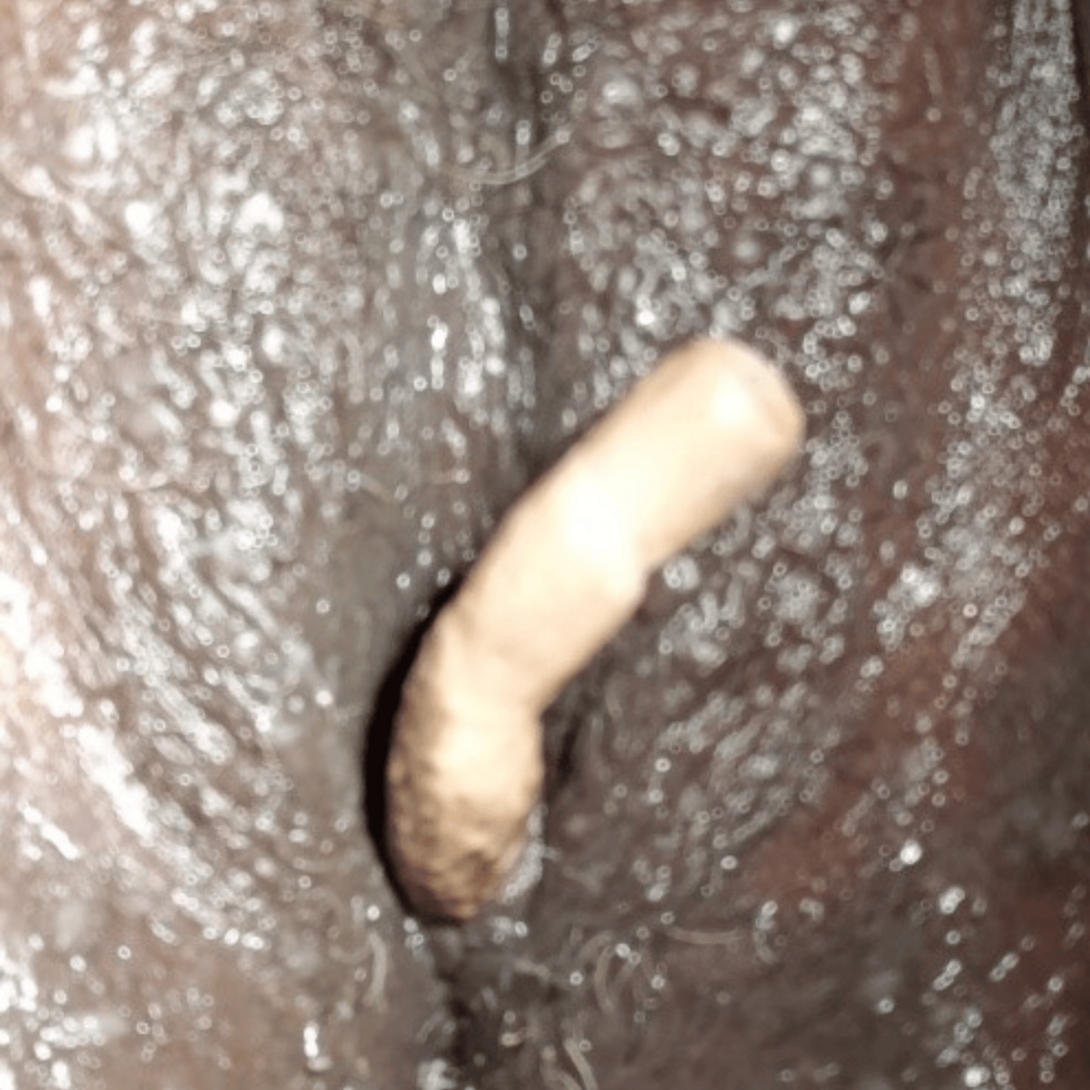
Image showing the severed calcified Foley catheter

**Figure 2:**
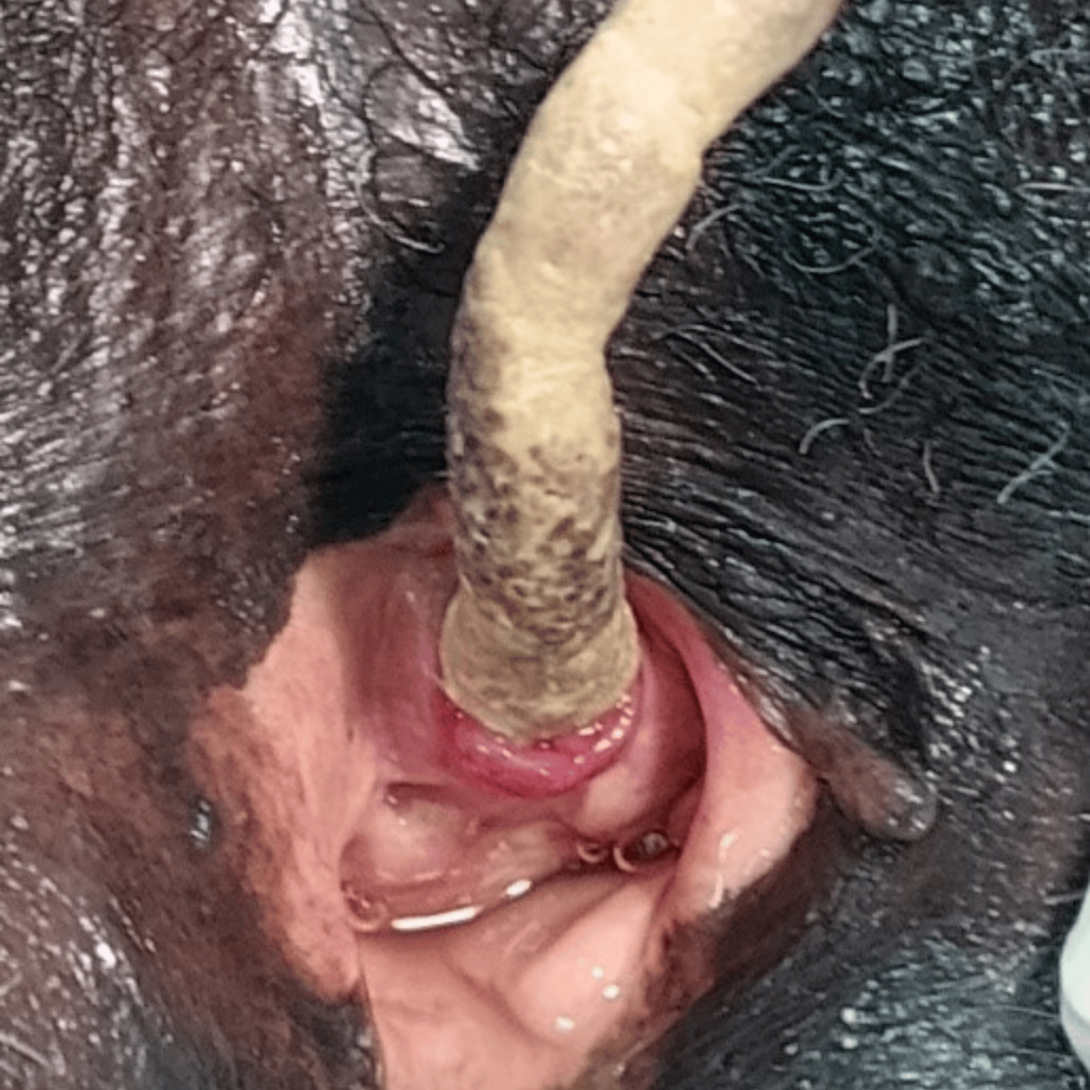
Foley catheter visible through the urethral meatus with urine draining into the vagina

**Figure 3:**
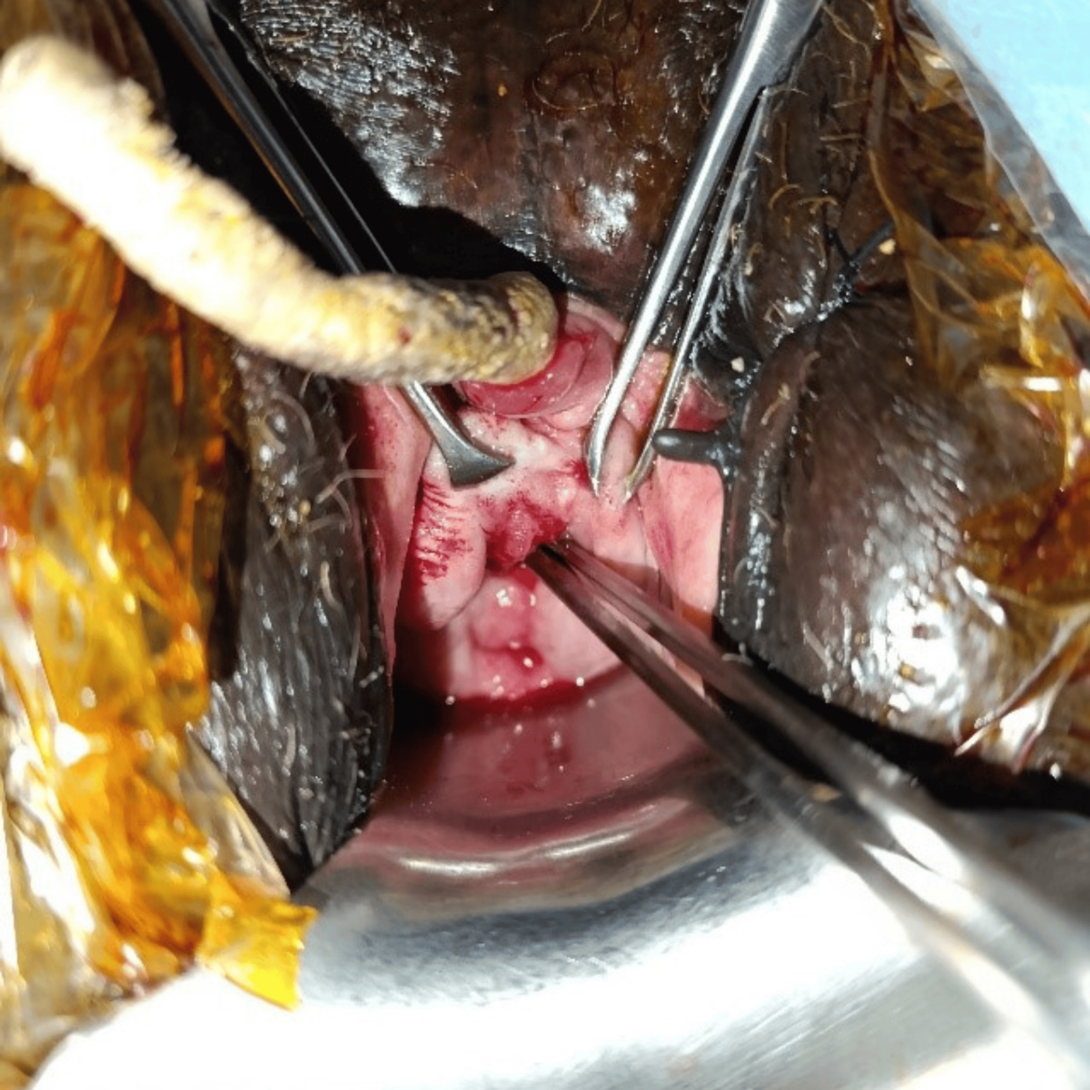
Intraoperative image: Exposure of the vesicovaginal fistula just above the cervix

On examination, the patient was in good general condition, and the hemodynamic parameters were stable. The urogenital examination showed a type 2 genital excision according to the WHO [6,7], an inflamed urethral meatus, with a segment of calcified probe visible at the level of the urethral meatus (Figures 1, 2), as well as a retro-trigonal juxta-cervico-uterine vesicovaginal fistula of approximately 2 cm (Figure 3).

No imaging studies were performed due to the obviousness of the clinical diagnosis and the context of care in a resource-limited setting.

**Figure 4:**
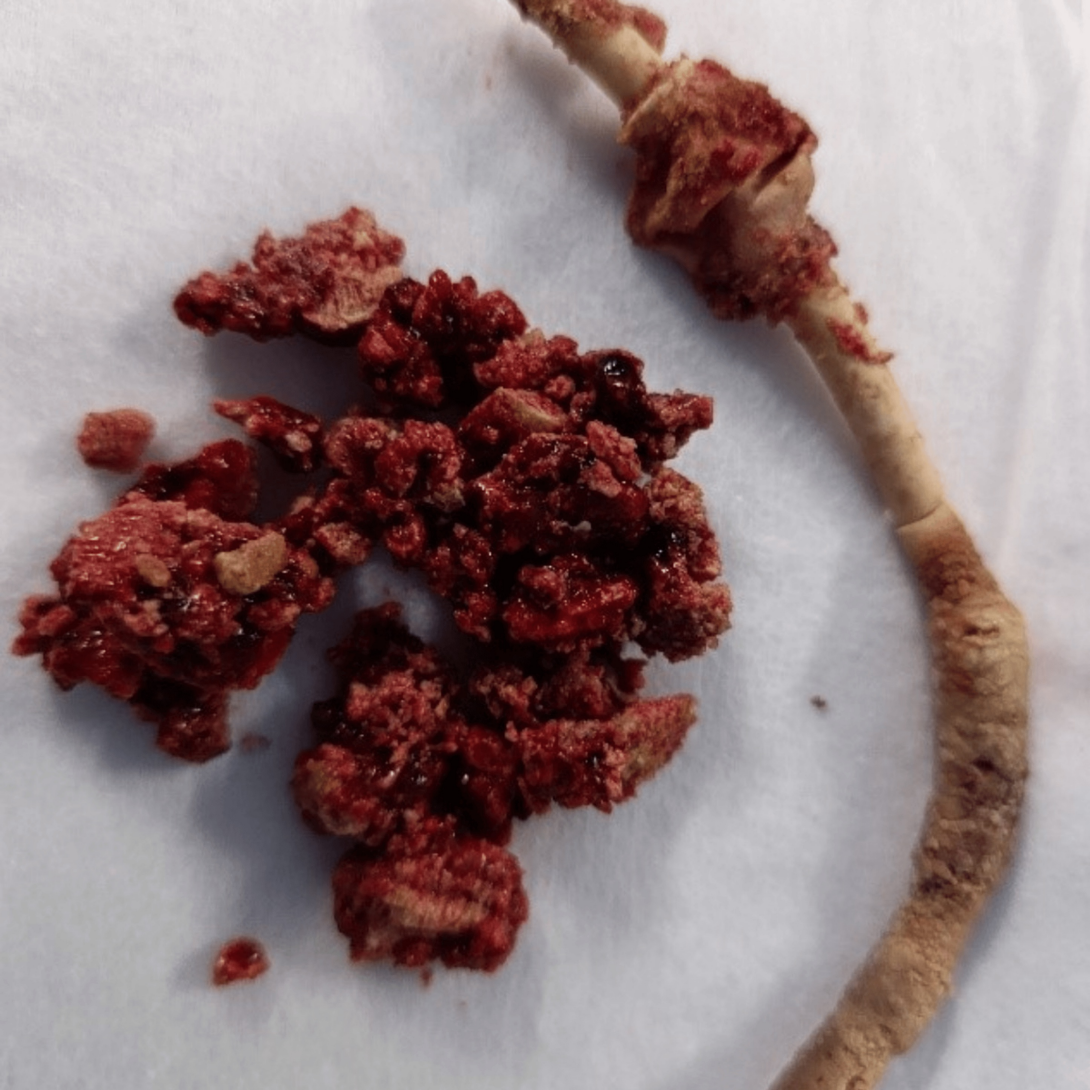
Postoperative image of the extracted Foley catheter tip along with the calculi

**Figure 5:**
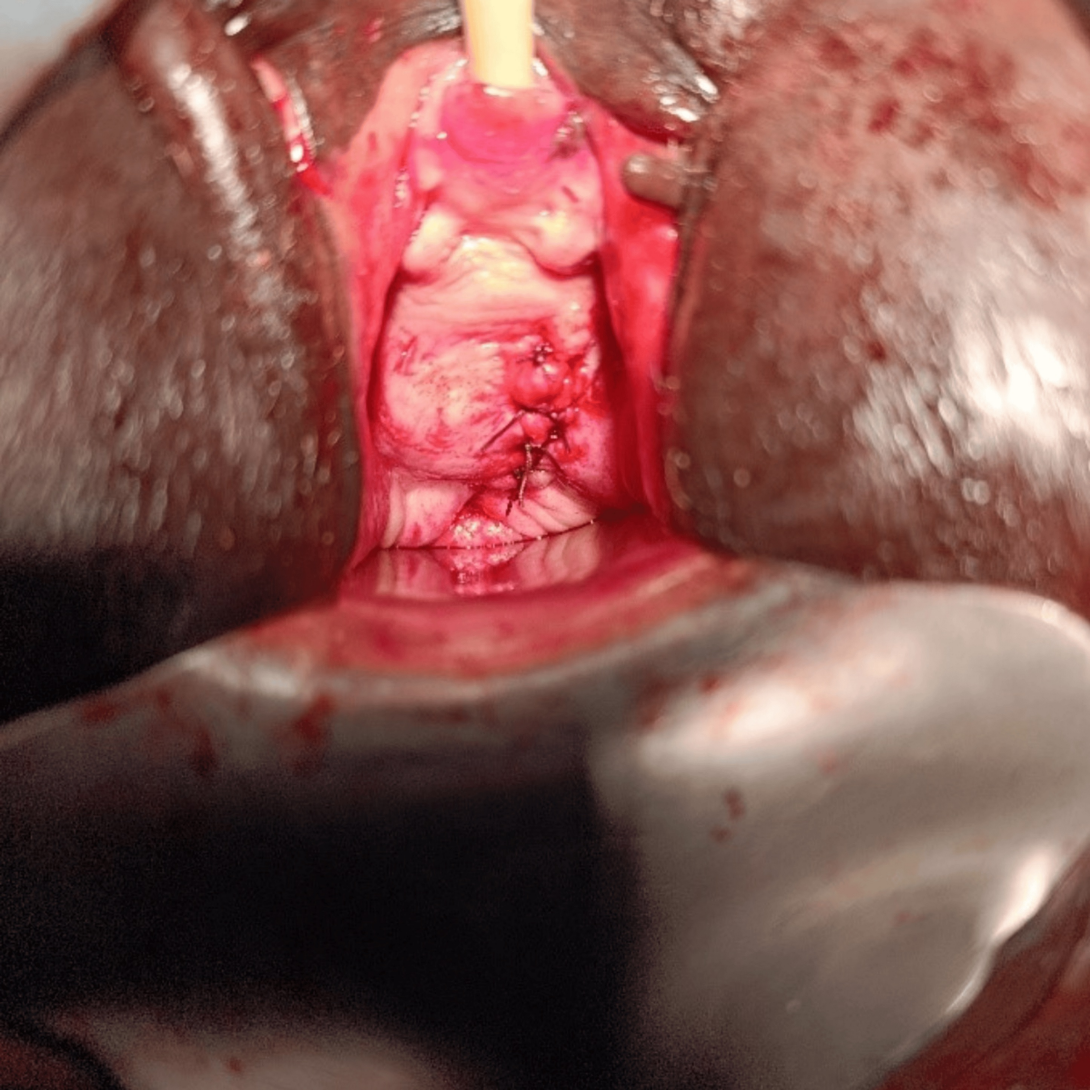
Postoperative image showing the end of fistula repair

The treatment consisted of sectioning the calcified distal portion of the catheter via the urethra. The proximal intravesical segment and the balloon calculi were extracted through the fistulous opening after fragmenting the calculi with forceps (Figure 4). Bladder irrigation with saline solution mixed with povidone-iodine was performed. The fistula was repaired vaginally during the same procedure (Figure 5). The postoperative course was uneventful; continence was restored on day 21 upon catheter removal. At the three- and six-month follow-ups, the patient was free of urinary incontinence. 

## Discussion

The mechanism of urinary catheter calcification in long-term catheter-bearing patients is primarily explained by the formation of a bacterial biofilm on the catheter surface. According to Stickler et al. [3,4], after four weeks, intravesical bacterial proliferation is observed, with the development of crystalline biofilms, favored by the presence of urease-positive bacteria such as *Proteus mirabilis*. These bacteria hydrolyze the urea in the urine into ammonia, leading to an increase in urinary pH and urine alkalinization. This phenomenon promotes the precipitation of struvite and calcium phosphate crystals, which are progressively deposited on the catheter and around the balloon.

At this stage, several complications can occur, including urinary tract infections, catheter obstruction, and bladder stone formation [8-10].

In our observation, the prolonged stay of the intravesical catheter fragment, associated with its progressive calcification, probably favored continuous mechanical and inflammatory aggression of the bladder wall. This situation may have led to progressive tissue damage, characterized by chronic inflammation, ischemia, and necrosis, resulting in the formation of a vesicovaginal fistula [5].

The delayed occurrence of urine leakage after catheter sectioning, initially around the catheter and then secondarily via the vagina, supports an acquired mechanism. However, the exact timing of the fistula's occurrence cannot be determined with certainty, and a preexisting undiagnosed fistula cannot be completely ruled out.

Clinically, patients may present with pelvic pain, recurrent urinary tract infections, urine leakage around the catheter, or catheter obstruction [9]. In some cases, the discovery is made during an unsuccessful attempt at removal, due to balloon adhesion to the bladder wall or failure to deflate. In our patient, access to the calcified catheter was facilitated by the presence of the fistulous opening.

The management of calcified catheters depends on the degree of encrustation and associated complications. Removal by simple traction can be difficult and may lead to urethral mucosal damage, significant bleeding, infection, or even urethral stricture.

In cases of large stone deposits or significant adhesion of the balloon to the bladder wall, surgical intervention may be necessary [10]. Bhandari et al. [11] reported the use of an open cystotomy for the extraction of a heavily encrusted catheter.

Ho et al. [12] reported a conservative approach: through the dilated opening of a cystostomy, they introduced a cystoscope with which they fragmented the calcifications around the balloon and, using forceps, removed the catheter via the urethra. They also described various minimally invasive management options, including endoscopic or percutaneous procedures that allow for fragmentation of the deposits and extraction of the catheter, although the techniques used may vary depending on the clinical situation.

In our patient, the intravesical segment was extracted directly through the fistulous opening, thus avoiding a complex procedure and allowing the fistula to be repaired during the same surgical session. This approach is an interesting feature of our case.

Several research studies have focused on developing urinary catheters capable of limiting the formation of bacterial biofilms. These studies rely on modifying the catheter surface to reduce bacterial adhesion, incorporating antimicrobial agents into the device material, or inhibiting the activity of urease-positive bacteria such as *Proteus mirabilis* [13-15]. While promising, these innovations remain largely inaccessible in routine practice.

## Conclusions

Calcification of Foley catheters is a known complication of prolonged urinary catheterization, but the occurrence of a vesicovaginal fistula in this context remains rare. Our observation highlights the formation of a vesicovaginal fistula secondary to the prolonged retention of an intravesical fragment of a calcified catheter. Extraction of the catheter fragment and calculi through the fistulous opening, followed by immediate vaginal repair, resulted in a good functional outcome. 

Preventing complications relies on regular monitoring, periodic replacement of catheters, and educating patients and their caregivers about the risks associated with prolonged urinary catheterization.
